# Religious activities, modern media use, and subjective wellbeing: a cultural goal consistency perspective

**DOI:** 10.3389/fpsyg.2026.1837670

**Published:** 2026-08-28

**Authors:** Haiting Wu, Jianhua Dai, Hanzun Li

**Affiliations:** Business School, China University of Political Science and Law, Beijing, China

**Keywords:** cultural goal consistency, moderating mechanism, modern media, religious activities, subjective wellbeing

## Abstract

**Introduction:**

In digital societies with evolving pathways to subjective well-being, modern media has become a new channel for individuals to seek cultural interaction and identity recognition beyond traditional religious methods. However, how these two mechanisms relate to subjective well-being across cultural contexts remains insufficiently understood.

**Methods:**

Using large-scale samples from the China General Social Survey and the European Social Survey, this study conducts regression analyses to examine the relationships among religious activities, modern media use, and subjective well-being.

**Results:**

The results indicate that (1) both religion and modern media function as channels connecting individuals with cultural meaning systems and significantly associated with subjective well-being; (2) the association between religious activities and subjective well-being across levels of modern media use: the positive association between religion and well-being is stronger in the Chinese sample with higher levels of media use, while this association is weaker in the European sample; and (3) the relationship between cultural connection mechanisms and subjective well-being varies according to cultural goal consistency.

**Discussion:**

This study contributes to the literature on religion, digital media, and well-being by highlighting the cultural conditions under which traditional and emerging meaning systems are associated with subjective well-being in increasingly digitalized societies.

## 1 Introduction

As economic recession, disease, and war continue to spread globally, individuals have become increasingly concerned about their personal happiness (Roth-Cohen et al., 2022). During periods of hardship, individuals often seek various outlets for comfort. Previous studies have demonstrated that higher subjective wellbeing (SWB) is associated with enhanced cognitive and social functioning (Batthyany and Russo-Netzer, 2014; Trzebiński et al., 2020). Challenging times often prompt individuals to seek meaning and reconnect with their inner selves, a process referred to as “quest culture” (Roof, 2001; Spännäri and Laceulle, 2021). Culture shapes the goals individuals pursue and is associated with the sources from which subjective wellbeing is derived (Diener et al., 1999). Religion has long been regarded as one of the most important cultural institutions linking individuals to collective values, moral norms, and existential meaning. Accordingly, regular participation in religious activities has consistently been identified as a significant predictor of subjective wellbeing (Shiah et al., 2016; Lim and Putnam, 2010). However, the mechanisms underlying this relationship remain insufficiently understood. Existing studies cannot fully explain why some individuals report higher levels of subjective wellbeing in relation to religious activities, whereas others experience only limited benefits. Similarly, the positive associations of religious participation and subjective wellbeing vary considerably across societies and cultural contexts (Kizilgeçit et al., 2025). These differences suggest that the relationship between religion and subjective wellbeing may depend not only on individual participation itself, but also on broader cultural environments and changing mechanisms of cultural connection.

The digital society has brought about a broader cultural environment and cultural connections (Shonfeld et al., 2021), providing a new perspective for understanding the relationship between religion and subjective wellbeing. Traditionally, religion functioned as one of the primary institutions connecting individuals with collective cultural values and systems of meaning. However, with the rapid expansion of the Internet and digital media, modern media increasingly serve as an alternative channel for cultural participation, identity construction, and emotional interaction. Individuals now spend substantial amounts of time in online environments, where modern media not only disseminates information but also is related to differences in value orientations, perceptions of belonging, and cultural goals (Cheshin et al., 2026). Consequently, religion and modern media are no longer independent factors associated with subjective wellbeing; instead, they increasingly interact as competing or complementary cultural connection mechanisms (Kulkarni et al., 2023).

Despite extensive research on religion and subjective wellbeing, two important limitations remain in the existing literature. First, most studies examine religious participation and media use separately, without considering how modern media may reshape the traditional cultural functions of religion in the digital era. Although previous research has confirmed that both religious participation and modern media use are associated with subjective wellbeing, limited attention has been given to the interaction between them. Second, prior explanations primarily focus on individual-level mechanisms such as social support, emotional coping, religious commitment, or religious attitudes (Keller et al., 2026), while paying insufficient attention to the broader issue of cultural goal alignment. As recent studies on religious attitudes suggest, the relationship between religion and wellbeing is deeply embedded within wider cultural and value structures. Consequently, existing studies cannot adequately explain why the wellbeing associations of religious activities vary substantially across individuals and societies. To address these issues, this study explores this phenomenon through the lens of cultural goal consistency, arguing that national, socioeconomic, educational, and age-related cultural backgrounds are related to variations in individuals’ cultural goals. Discrepancies between individuals’ cultural goals and religious cultural goals may be associated with differences in individuals’ sense of belonging and achievement upon attaining religious cultural goals, as well as their attitudes toward experiences, thereby affecting the extent to which religious activities are associated with subjective wellbeing. Accordingly, we conducted separate analyses using culturally distinct samples to examine the role of cultural goal consistency.

This exploratory study makes three primary contributions. First, this study extends the literature on religion and subjective wellbeing by incorporating modern media into the analysis of cultural pathways to wellbeing. By comparing China and European societies, this study provides cross-cultural evidence on how traditional religious practices and emerging digital media are associated with subjective wellbeing under different social and cultural conditions. Second, this study develops the concept of “cultural goal consistency” to explain the heterogeneity of religion–wellbeing associations across cultural contexts. Rather than assuming a universal association between religious activities and wellbeing, this framework emphasizes that the observed linkage between religion and subjective wellbeing varies according to the compatibility of religious values, media-related cultural orientations, and broader social value systems. Third, by integrating religion, modern media, and cultural context into a unified analytical framework, this study contributes to the international literature on religion, digitalization, and wellbeing. The findings suggest that digital transformation does not necessarily displace traditional meaning systems; instead, the associations involving these systems differ across specific cultural environments.

## 2 Review and hypothesis

### 2.1 Theoretical basis

Theories explaining happiness primarily include genetic theory, personality trait theory, personality-environment interaction theory, comparative theory, adaptation and coping theory, needs theory, and culture- and goal-related theory. Specifically regarding the theories concerning the relationship between culture and happiness, they can be broadly classified into two main branches.

Theories concerning the relationship between culture and wellbeing can be broadly categorized into two primary branches: culture-goal theories and needs theories. Culture-goal theories have garnered significant attention in recent years, conceptualizing culture as an environmental factor that is associated with subjective wellbeing. These theories posit that the cultural environment in which individuals live is related to subjective wellbeing through cultural goals. Subjective wellbeing tends to be higher when individuals are able to achieve, or anticipate achieving, these goals; conversely, it tends to be lower when such goals are unattainable. Oishi et al. (1999) argue that subjective wellbeing increases when individuals achieve goals highly valued by their culture or subculture. Dursun and Cesur (2016) found, using Turkish data, that compulsory education significantly enhances women’s subjective wellbeing, whereas for men, higher education raises expectations, and mismatches between achievement and expectations lead to declines in subjective wellbeing. Evrensel (2015) found that economic freedom was positively associated with subjective wellbeing in predominantly Christian countries, whereas a negative association was observed in predominantly Muslim or Buddhist countries. Demand theory posits that cultural consumption and cultural experiences satisfy individual needs, thereby enhancing subjective wellbeing. Lee and Heo (2021) argues that cultural activities satisfy psychological needs through aesthetic value, thereby enhancing happiness. Wheatley and Bickerton (2019) argue that cultural activities and cultural consumption fulfill five psychological needs—transcendence-relaxation, autonomy, mastery, meaning, and belonging—thereby enhancing happiness. This study undertakes theoretical exploration based on culture–goal theory as it simultaneously explains the association between modern media and variations in subjective wellbeing across different cultural contexts.

### 2.2 Religious activities and subjective wellbeing

Against the backdrop of escalating global crises, individuals are increasingly seeking reconnection with cultural meaning in their pursuit of subjective wellbeing, a process facilitated through two primary channels: religion and modern media. Religion, as the traditional core cultural channel, is related to individuals’ cultural goals through three mechanisms—shared rituals, moral norms, and community bonds—thereby being associated with subjective wellbeing. First, shared rituals may generate emotional synchrony and collective identity, thereby strengthening meaning construction, social cohesion, and subjective wellbeing (Páez et al., 2015). Second, the internalization of religious moral norms may enhance self-regulation by promoting behavioral consistency and stable value orientations, thereby being associated with differences in subjective wellbeing (McCullough and Willoughby, 2009). Third, religious communities often provide durable social networks and interpersonal trust, which strengthen social integration and emotional support (Lim and Putnam, 2010). Through these mechanisms, religion functions not merely as a belief system but as a cultural framework associated with individual goals, identity, and wellbeing. This leads to hypothesis H1:

*H1*: Religious activity is positively associated with individuals’ subjective well-being.

### 2.3 Religious activities, modern media and subjective wellbeing

Modern media, represented by Internet technology, functions as a contemporary cultural channel that is associated with differences in individuals’ cognition, emotions, and behavior through acculturation effects, thereby relating to variations in their cultural goals. Recent studies increasingly emphasize that modern media can contribute to subjective wellbeing by facilitating social connection, emotional support, and identity expression in digital environments. Digital platforms enable individuals to maintain interpersonal relationships and participate in online communities, thereby strengthening social connectedness and improving psychological wellbeing (Keles et al., 2020). Similarly, Liu et al. (2018) demonstrate that active social media engagement is positively associated with wellbeing because online interactions provide users with opportunities for self-expression, social support, and interpersonal belonging. Beyond interpersonal mechanisms, digital media also expand individuals’ access to informational and cultural resources, which can enhance life satisfaction by increasing perceived social inclusion and personal agency (Irmer and Schmiedek, 2023). Furthermore, recent research highlights that the positive effects of modern media are particularly evident when usage patterns are oriented toward meaningful communication rather than passive consumption, suggesting that digital media function as important social and cultural resources that support subjective wellbeing (Meier and Reinecke, 2021). Collectively, these findings indicate that modern media serve not merely as technological tools but as cultural connection channels associated with individuals’ subjective wellbeing. This leads to hypothesis H2:

*H2*: Modern media use is positively associated with individuals’ subjective well-being.

Early research on religion and modern media primarily focused on whether the expansion of modern media would weaken religious authority and accelerate secularization. From the perspective of modernization theory, economic development, scientific advancement, and technological transformation were expected to reduce the social significance of religion (Stark, 1999). Pesando et al. (2025) showed that digital technologies are associated with changing patterns of cultural participation and value orientations. Similarly, Campbell and Tsuria (2022) proposed that the Internet may challenge traditional religious systems by disseminating alternative value orientations, weakening centralized religious authority, and transforming forms of religious community participation. However, subsequent digital religion research has challenged the assumption that digitalization necessarily leads to religious decline. Rather than simply replacing religion, modern media may reshape the forms through which religious meanings are produced and experienced (Evolvi, 2022). For example, social media enables religious groups to expand communication networks, create online communities, and provide emotional support beyond traditional offline institutions (Adeoye and Noorhayati, 2024).

These findings suggest that modern media is not merely associated with variations in religious participation patterns, but may also correspond to differences in how religious activities are related to individuals’ psychological wellbeing. Specifically, by providing alternative cultural resources or reinforcing existing value systems, modern media may be associated with differences in the extent to which religious activities relate to individuals’ subjective wellbeing (Evolvi, 2022).

Modern media may therefore be related to variations in the association between religious activities and subjective wellbeing through a conditional cultural mechanism. In some contexts, online religious interaction may enhance emotional support, collective identity, and value affirmation associated with religious practice (Besika, 2023). Therefore, the strength of this association may vary depending on the degree of cultural goal consistency between online media culture and religious culture. When media environments provide value orientations inconsistent with individuals’ religious goals, the psychological benefits associated with religious activities may decrease (Beyens et al., 2024). Conversely, when online culture reproduces or reinforces religious values and identity, modern media may be associated with a stronger relationship between religious participation and wellbeing through complementary cultural mechanisms (Meier and Reinecke, 2021). Accordingly, modern media may correspond to differences in the relationship between religious activities and subjective wellbeing. Thus, this study proposes the following hypothesis:

*H3*: The association between religious activities and subjective well-being varies with modern media use.

#### 2.3.1 Cultural goal consistency and cultural differences

Cultural differences add further complexity to this mechanism. Drawing on Sellin’s (1938) cultural conflict perspective, this study argues that individuals may experience psychological consequences when different cultural value systems provide inconsistent normative expectations. Building on this perspective, this study introduces cultural goal consistency, which refers to the degree to which the cultural goals embedded in different social systems, such as religion, modern media, and mainstream social values, are mutually compatible with individuals’ internalized value orientations. Furthermore, cultural goal consistency may vary across different levels of social structure. Specifically, cultural differences span macro-level regional and ethnic variations, meso-level group and class cultural variations, and micro-level temporal cultural variations.

At the macro level, since there are numerous ethnic groups (a total of 143) in Europe and China, a detailed discussion would be overly lengthy. Therefore, this study does not explore the ethnic cultural differences at the macro level but focuses on variations associated with national cultural differences. Although substantial differences exist among Nordic, Western European, Southern European, and Eastern European countries in terms of religious institutions, public religiosity, and the cultural role of religion, religion has continued to function as an important source of regional identity and collective value orientation (Casanova, 1994). In comparison, within the Chinese context, religious culture has been more socially embedded in local communities and interpersonal networks rather than operating primarily as an overarching public institution (Fei et al., 1992; Yang, 2011). Religion may occupy different cultural positions across different national settings, thereby corresponding to differences in the relationship between religious activities and subjective wellbeing in distinct ways. This leads to the following hypothesis:

*H4a*: The association between religion, modern media, and subjective well-being varies across national cultural contexts.

At the meso level, class-level and group-level cultural differences are employed to illustrate the importance of cultural goal consistency. First, cultural differences are reflected in stratified group-based value orientations associated with socioeconomic positioning. Income-based stratification is employed as a proxy for socioeconomic cultural differences, as economic resources shape exposure to distinct social networks, lifestyle norms, and evaluative standards, thereby corresponding to variation in the degree of alignment between individual goals and religious cultural goals. In this sense, class culture reflects differentiated cultural goal environments within the same macro-institutional context. This leads to the following hypothesis:

*H4b*: The association between religion, modern media, and subjective well-being varies across socioeconomic cultural contexts.

At the same meso level, given the broad scope of group-level cultural differences, we use educational cultural differences to represent this dimension. Education not only transmits skills but also is associated with differences in cognitive schemas and authority orientations, thereby relating to individuals’ receptivity to religiously grounded value systems (Norris and Inglehart, 2011). The value objectives of meso-level religious culture may correspond differently with the objectives of micro-level, era-specific culture. Consequently, educational differences capture variation in the internal consistency between secular rationalized value systems and religious cultural goals. Therefore, the following hypothesis can be proposed:

*H4c*: The association between religion, modern media, and subjective well-being varies across educational cultural contexts.

At the micro level, age is employed as a proxy for life-course–based temporal cultural variation. Individuals born in different historical periods are exposed to distinct institutional environments and cultural narratives, leading to cohort-based differences in value internalization. Differences in micro-level, era-specific cultures are associated with variations in the extent to which individuals internalize religious culture. These temporal cultural differences may correspond to differences in the stability and adaptability of cultural goal consistency across religious participation and media use. Therefore, the following hypothesis can be proposed:

*H4d*: The association between modern media, religion, and subjective well-being varies across age-related cultural contexts.

Furthermore, in comparison with religious cultures, the material disseminated through modern media is more readily shaped by macro-level (mainstream) culture, and such influence occurs in real time(Couldry and Hepp, 2018). For example, with respect to regional cultural differences, the extent to which national mainstream culture aligns with religious values is associated with differences in the way modern media relates to religious values (Lövheim, 2011). In societies characterized by strong religious embeddedness, modern media may serve to extend religious values; whereas in challenging traditional religious interpretations or reducing the relative centrality of religious frameworks in meaning construction.

**Figure 1:**
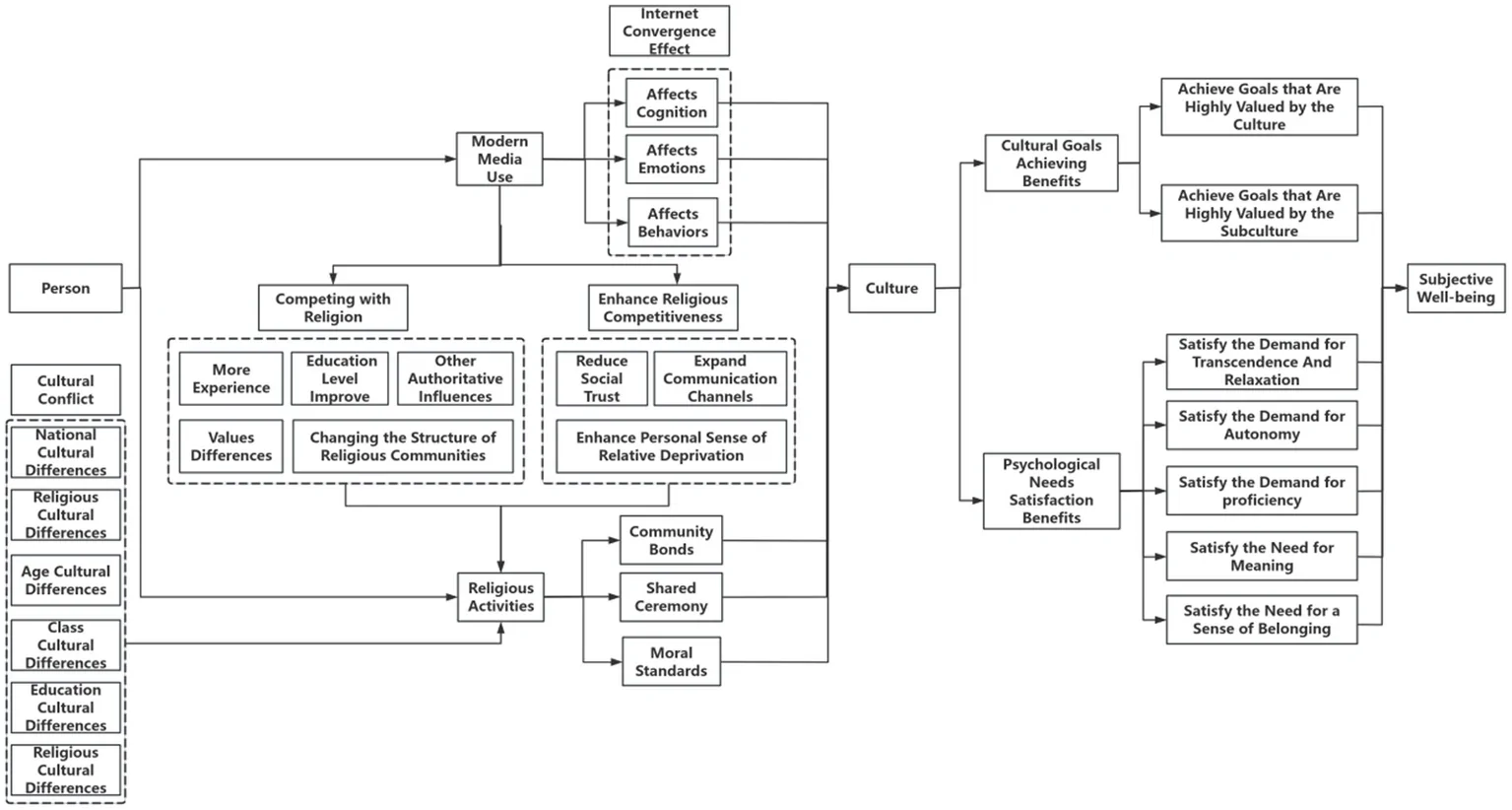
Relationship between religion, Internet use and subjective wellbeing.

The foregoing mechanisms indicate that the relationship between modern media, religion and subjective wellbeing resides in the competition and cooperation of cultural connection patterns. Individual subjective wellbeing may depend not only on the intensity of participation but also on the consistency of the cultural goals transmitted through the two channels. This dynamic adaptation process may help explain the individual differences in the wellbeing benefits derived from religious activities, as shown in Figure 1.

## 3 Data, variables and methods

### 3.1 Dataset construction

This study combines data from the China General Social Survey (CGSS) and the European Social Survey (ESS) to examine the relationships among religion, media use, and subjective wellbeing across two culturally distinct social contexts. After examining missing data patterns across the variables included in the regression models, observations with missing values were excluded using listwise deletion. The final Chinese sample consisted of 4,805 valid observations from 31 provinces in mainland China, and the final European sample retained 21,950 valid observations from 39 countries. Given the temporal mismatch between the latest available GSS data in the United States (2022/2024) and the target year (2023), we exclude GSS data to avoid cross-year comparison biases arising from cyclical fluctuations. Given that large-scale social surveys commonly measure psychological constructs using ordinal scales, these variables are treated as approximately continuous following established practices in behavioral and social science research (Norman, 2010; Rhemtulla et al., 2012). Log transformations were applied to reduce skewness and mitigate the influence of extreme observations, thereby improving the statistical properties of regression models (Wooldridge, 2025).

To ensure the reliability of regression estimates, several diagnostic tests were conducted. First, variance inflation factor (VIF) tests were performed to assess multicollinearity, with all values below the recommended threshold. Second, model fit was evaluated using R-squared, adjusted R-squared, and F-statistics. Third, residual diagnostics, including heteroskedasticity tests and residual distribution assessments, were conducted. Robust standard errors were applied in regressions to ensure reliable statistical inference.

### 3.2 Research methods

Based on the dual-path framework of cultural connection and prior research on subjective wellbeing, religion, and media acculturation, this study conceptualizes subjective wellbeing as being associated with religious activities, modern media use, and other contextual factors. Accordingly, the following general analytical framework is specified in Equation 1:


SWB=f(RA,IA,Z) (1)

where *SWB* represents subjective wellbeing, *SWB* denotes subjective wellbeing, *RA* represents religious activities, *IA* represents modern media use, and *Z* denotes other explanatory factors derived from wellbeing theories.

The above model does not allow examination of variations across different cultural contexts and cannot adequately verify the relationship between religious activities and modern media use. Therefore, a multi-stage association analysis framework was employed to examine the interactive relationships among religious activities, modern media use, and subjective wellbeing.

To examine the association between religious activities and subjective well-being, the following baseline model was specified in Equation 2:


SWB=α+βRA+μ∑Z+ε (2)

To examine whether modern media use conditions the association between religious activities and subjective well-being, the following model was specified in Equation 3:


$$\begin{array}{c} \mathrm{SWB}=\alpha +\mathrm{\beta RA}+\mathrm{\gamma IA}+\mathrm{\theta RA}\cdot \mathrm{IA}+\mu \sum Z+\varepsilon \end{array}$$ (3)

Finally, based on the theoretical analysis of cultural differences, we propose a media-culture goal integration framework. Modern media may be associated with differences in the relationship between religious activities and subjective wellbeing by providing alternative cultural resources and expanding religious communication channels. However, this relationship may vary according to the degree of compatibility between individuals’ cultural goals and religious cultural goals. Accordingly, the improved model is expressed as Equation 4:


$$\begin{array}{c} \mathrm{SWB}=f(\mathrm{RA},\mathrm{IA},\mathrm{CC},Z) \end{array}$$ (4)

where *CC* denotes cultural goal consistency, may vary across different social and cultural contexts, including national, class, educational, and generational cultural differences. Therefore, this study examines the heterogeneous associations between religious activities, modern media use, and subjective wellbeing across different population groups to indirectly explore the implications of cultural goal consistency.

### 3.3 Variables

#### 3.3.1 Religious activities

The measurement of religious activities must account for their cultural embeddedness and the diversity of behavioral expressions. This study, based on the cultural context of two large databases and their questionnaire design, employed different items and scoring schemes to capture the intensity of individual religious participation, with the aim of obtaining comparable measurement results across cultural settings.

In the Chinese sample, religious activity was measured using the CGSS question: “How frequently do you participate in religious activities?” Following the official CGSS coding scheme, responses range from 1 (“Never participated”) to 9 (“Several times a week”), with higher values indicating more frequent participation.

In the European sample, as participation in religious ceremonies is closely related to religious festivals, daily life events (e.g., weddings and funerals), and public services, the frequency of attendance alone may not fully capture an individual’s religious commitment. So religious activities was measured using the ESS question: “Apart from when you are at religious services, how often, if at all, do you pray?” Responses range from 1 (“Never”) to 7 (“Every day”).

#### 3.3.2 Modern media use

This study employed standardized questionnaire designs from the CGSS and ESS to assess the intensity of individual modern media usage.

In the Chinese sample, respondents were asked: “In the past year, how often have you used the following media?” This question encompassed dimensions such as “using the Internet via smartphones, computers, tablets, and other smart devices.” Following CGSS coding, responses range from 1 (“Never”) to 5 (“Very frequently”).

In the European sample, modern media use was measured through the ESS question: “How often do you use the Internet on these or any other devices, whether for work or personal use?” Responses range from 1 (“Never”) to 5 (“Every day”). Higher values represent greater frequency of modern media use.

#### 3.3.3 Subjective wellbeing

Subjective wellbeing was measured using happiness indicators available in CGSS and ESS. Because happiness scales differ between surveys, the analyses retain the original coding schemes.

In the Chinese sample, subjective wellbeing was measured by the CGSS question: “Overall, do you feel that your life is happy?” Responses range from 1 (“Very unhappy”) to 5 (“Very happy”).

In the European sample, subjective wellbeing was measured by the ESS question: “Taking all things together, how happy would you say you are?” Responses range from 0 (“Extremely unhappy”) to 10 (“Extremely happy”). Higher values indicate higher self-reported levels of subjective wellbeing.

#### 3.3.4 Control variables

Based on culture-goal theory and needs theory, this study includes several control variables to account for potential differences across respondents within each cultural context. These variables include gender (GEN), educational attainment (EDU), income (IC), political participation (PP), health status (SH/OH), social interaction (SS/OS), and working hours (WT). These variables are constructed according to the official coding schemes of CGSS and ESS. All the questions corresponding to these variables were designed to account for characteristic differences between Chinese and European respondents. Importantly, although the exact operationalization of some control variables differs across datasets, the underlying theoretical constructs remain consistent. The detailed coding procedures are reported in Tables 1, 2.

**Table 1:** Questions, options and assignments of control variables (China).

Variable	Question	Options and value assigned													
		1	2	3	4	5	6	7	8	9	10	11	12	13	14
Gender	Respondent’s gender	Male	Female												
Educational attainment	What is your highest level of education?	No education	Private tutoring	Literacy classes	Elementary school	Junior high school	Vocational high school	Regular high school	Secondary vocational school	Technical school	Associate degree (adult higher education)	Associate degree (regular higher education)	Bachelor’s degree (adult higher education)	Bachelor’s degree (regular higher education)	Graduate degree or higher
Income	What was your total income (in yuan) for the entire year of 2022?														
Political participation	What is your current political affiliation?	Masses	Communist Youth League member	Democratic party member	Communist Party member										
Subjective health	How do you perceive your current physical health status?	Very unhealthy	Somewhat unhealthy	Average	Somewhat healthy	Very healthy									
Objective health	In the past 4 weeks, how frequently have health issues affected your work or other daily activities?	Always	Often	Sometimes	Rarely	Never									
Social interaction	Over the past 4 weeks, how often have you felt lonely?	Never	Rarely	Sometimes	Often	Very often									
Objective social assessment	In the past year, have you frequently engaged in the following activities during your free time—socializing/ visiting others?	Never	Rarely	Sometimes	Often	Very frequently									
Working hours	When you have a job, how many hours do you typically work per week, including overtime?														

**Table 2:** Questions, options and assignments of control variables (Europe).

Variable	Question	Options and value assigned									
		1	2	3	4	5	6	7	8	9	10
Gender	Which of the options on this card best describes you?	Male	Female								
Educational attainment	About how many years of education have you completed, whether full-time or part-time?										
Income	Using this card, please tell me which letter describes your household’s total income, after tax and compulsory deductions, from all sources?	J	R	C	M	F	S	K	P	D	H
Political participation	Have you donated to or participated in a political party or pressure group?	No	Yes								
Subjective health	How is your health in general?	Very bad	Bad	Fair	Good	Very good					
Objective health	Are you hampered in your daily activities in any way by any longstanding illness, or disability, infirmity or mental health problem?	Yes, a lot	Yes, to some extent	No							
Subjective loneliness perception	How much of the time during the past week did you feel this wayyou felt lonely?	None or almost none	Sometimes	Most of the time	All or almost all of the time						
Objective social assessment	Compared to other people of your age, how often would you say you take part in social activities?	Much less than most	Less than most	About the same	More than most	Much more than most					
Working hours	Regardless of your basic or contracted hours, how many hours {do/did} you normally work a week (in your main job), including any paid or unpaid overtime										

## 4 Results

### 4.1 Benchmark regression results

The results of the baseline regression are presented in Table 3. Results (1) and (3) correspond to models estimated without control variables, while Results (2) and (4) correspond to models estimated with control variables. Religious activities are significantly positively associated with subjective wellbeing, thereby supporting Hypothesis H1. Religion functions as a “channel connecting individuals to culture,” being associated with higher levels of wellbeing through systems of meaning and cultural belonging within specific social contexts. Compared with the European sample (estimated coefficient is 0.0118), the estimated coefficient of religious activities is relatively larger in the Chinese sample (estimated coefficient is 0.0608), indicating a stronger association between religious activities and subjective wellbeing in this context. This difference may reflect variations in the social and cultural environments in which religious activities are embedded. In the Chinese context, rapid socioeconomic transformation has occurred alongside changes in community structures and social relationships. For some individuals, religious activities may be associated with sources of meaning and a sense of collective belonging, which may correspond to lower levels of perceived meaning loss during periods of rapid change. This finding is consistent with Campante and Yanagizawa-Drott (2015)—in regions with inadequate social security, the association between religion’s community integration function and happiness is more pronounced.

**Table 3:** Benchmark regression results.

Variable name	China				Europe			
	Result(1)		Result(2)		Result(3)		Result(4)	
	Coeff.	*t*	Coeff.	*t*	Coeff.	*t*	Coeff.	*t*
Intercept term	1.5577***	84.49	1.0812***	29.77	2.1315***	378.9	2.2486***	108.49
LN_RA	0.0457***	0.044	0.0608***	2.78	−0.001	−0.31	0.0118***	3.96
GEN			0.0555***	4.8			0.0212***	6.79
EDU			0.004*	1.87			0.0012***	2.86
IC			0.0000***	3.01			0.0077***	12.12
PP			0.0349***	5.6			0.0103*	1.79
SH			0.0528***	7.82			0.0505***	23.32
OH			0.0199***	3.25			0.0074**	2.2
SS			−0.0212***	−3.76			−0.1118***	−45.54
OS			0.032***	6.32			0.026***	14.98
WT			−0.0002	−0.25			0.0012***	2.86
*R* ^2^	0.0008		0.0826		0.0000		0.1783	

LN_RA denotes the natural logarithm of religious activities; LN_IA denotes the natural logarithm of modern media use; LN_RP denotes the natural logarithm of religious piety. * indicates *p* < 0.1, ** indicates *p* < 0.05, *** indicates *p* < 0.01. The same variable definitions apply to Tables 4–7.

### 4.2 Robustness test

#### 4.2.1 Setting instrumental variables

Religious devotion was introduced as an instrumental variable for religious activities to examine whether potential endogeneity affects the baseline estimates. The rationale is twofold: (1) devotion, as a deep-seated faith attitude, is expected to be closely related to the intensity of religious participation while not being directly associated with subjective wellbeing after accounting for religious activities; (2) in cross-sectional data, religious devotion reflects a stable, long-term faith identity that is less likely to vary with short-term emotional fluctuations, thereby reducing concerns regarding the possibility that differences in subjective wellbeing are associated with differences in devotion.

This study used RP to represent the degree of religious devotion. For the Chinese sample, religious devotion was derived from the question: “Are you actively involved in the activities of a religious organization?” Response options range from 1 to 3, where 1 means “Yes, I am a member and actively participate” and 3 “Not a member.” For the European sample, religious devotion was measured by the question: “Except for special occasions, how often do you attend religious ceremonies?” This question contained 11 response options (0–10), with 0 indicating no devotion and 10 indicating completely devotion.

The regression results estimated using the two-stage least squares (2SLS) approach are presented in Table 4. As shown in columns (1)–(4), after addressing potential endogeneity concerns through the instrumental variable approach, the estimated coefficient of religious activities remains significantly positive, indicating a robust positive association between religious activities and subjective wellbeing.

**Table 4:** Robustness test results.

Variable name	Setting instrumental variables				Using survey weights				Removal of outliers	
	China		Europe		China		Europe		Europe	
	Coeff.	*z*	Coeff.	*z*	Coeff.	*t*	Coeff.	*t*	Coeff.	*t*
Intercept term	1.0496***	21.32	2.0062***	59.54	1.0237***	23.34	1.9861***	55.84	2.2452***	107.25
LN_RP	0.1167**	2.53	0.0202**	2.35	0.0546**	2.45	0.0211**	2.22	0.0115***	3.80
GEN	0.0505***	4.32	0.0203***	4.85	0.0545***	4.17	0.0216***	4.97	0.0216***	6.85
EDU	0.0026	1.27	0.0098***	2.89	0.0069	0.67	0.0115***	3.15	0.0012***	2.85
IC	0.0000***	3.23	0.0305***	12.67	0.0494***	5.98	0.0276***	10.91	0.0078***	12.14
PP	0.0358***	6.09	−0.0218***	−2.87	0.0310***	4.59	−0.0210**	−2.58	0.0096*	1.65
SH	0.0524***	7.44	−0.0554***	−17.37	0.0516***	6.74	−0.0541***	−16.24	0.0506***	23.13
OH	0.0196***	2.99	0.0163***	3.22	0.0185**	2.59	0.0200***	3.81	0.0076**	2.25
SS	−0.0221***	−3.58	−0.1226***	−26.49	−0.0173**	−2.56	−0.1245***	−25.06	−0.1130***	−45.52
OS	0.0306***	5.73	0.0283***	11.37	0.0328***	5.71	0.0295***	10.98	0.0264***	15.04
WT	−0.0002	−0.24	−0.0005***	−2.86	0.0000	0.03	−0.0004**	−2.37	−0.0003**	−2.51
*R* ^2^					0.0897		0.1344		0.1805	

#### 4.2.2 Using survey weights

To further ensure the robustness of the baseline findings, additional weighted regressions were conducted using survey-specific weighting schemes for both datasets. For the ESS sample, the official design weight (dweight) was applied to account for unequal selection probabilities. For the CGSS sample, the integrated weight, which adjusts for multistage sampling and post-stratification, was employed to ensure national representativeness. The results in columns (5)–(8) of Table 4 remain consistent with the baseline estimations: the core explanatory variable retains a statistically significant positive association with subjective wellbeing across specifications with and without control variables. These findings provide further support that the observed associations are robust to alternative weighting specifications.

#### 4.2.3 Removal of outliers

War-related contexts may represent distinctive social environments that differ from other countries in terms of perceived security and social conditions. Accordingly, we excluded data from countries engaged in armed conflict in 2022 as a robustness check. Among the countries included in the European Social Survey, Ukraine was involved in the Russia-Ukraine war in 2022, and Israel and Palestine were in conflict. Consequently, the data for Ukraine and Israel were excluded, and the regression for European countries was re-estimated, with the results presented in columns (9) and (10) of Table 4. The regression results remain robust, with religious activities retaining a significant positive association with subjective wellbeing.

### 4.3 Moderation analysis

This study first explores the associations between media usage and religious activities. The results are presented in Table 5. In both China and Europe, religious activities and modern media use are significantly positively associated with subjective wellbeing. Moreover, the estimated coefficients of modern media use are larger than those of religious activities in both samples, suggesting that the association between modern media use and subjective wellbeing is relatively stronger than that of religious activities. These findings provide support for Hypothesis H2.

**Table 5:** Media use and religious activities.

Variable name	China		Europe	
	Coeff.	*t*	Coeff.	*t*
Intercept term	1.0255***	23.11	2.1186***	38.72
LN_RA	0.0578***	2.65	0.0121***	4.07
LN_IA	0.1802***	5.51	0.0980***	3.39
GEN	−0.0564***	−4.90	−0.0212***	−6.77
EDU	0.0009	0.40	0.0011***	2.61
IC	0.0000***	2.78	0.0076***	11.94
PP	0.0306***	4.88	0.0105*	1.82
SH	0.0507***	7.53	0.0501***	23.15
OH	0.0185***	3.03	0.0074**	2.22
SS	−0.0203***	−3.60	−0.1115***	−45.44
OS	0.0281***	5.51	0.0261***	15.03
WT	−0.0001	−0.15	−0.0003***	−2.67
*R* ^2^	0.0884		0.1787	

To further examine whether modern media use conditions the relationship between religious activities and subjective wellbeing, we introduce the interaction term between media use and religious activities into the regression models. The results are presented in Table 6.

**Table 6:** Media use, religious activities and subjective wellbeing.

Variable name	China		Europe	
	Coeff.	*t*	Coeff.	*t*
Intercept term	1.1184***	30.36	1.3478***	5.82
LN_RA	0.0600***	3.05	0.3015**	2.39
LN_IA	−0.0068	−1.50	0.1371***	2.92
c.LN_RA#c.LN_IA	0.0229*	1.75	−0.0586**	−2.28
GEN	0.0540***	4.63	0.0202***	4.88
EDU	0.0036	1.62	0.009***	2.66
IC	0.0000***	3.43	0.0301***	12.65
PP	0.0350***	5.99	−0.0223***	−2.90
SH	0.0532***	7.48	−0.0547***	−18.77
OH	0.0206***	3.13	0.0163***	3.64
SS	−0.021***	−3.42	−0.1222***	−36.93
OS	0.0317***	5.93	0.0283***	12.16
WT	−0.0002	−0.22	−0.0004***	−3.16
*R* ^2^	0.0859		0.1359	

As shown in Table 6, the coefficient of the interaction term between media use and religious activities is significantly positive in the Chinese sample, whereas it is significantly negative in the European sample. So the Hypothesis H3 is supported.

In the Chinese sample, the relationship between religious activities and subjective wellbeing is stronger among individuals with higher levels of media use. This pattern may suggest that religious activities are often embedded in informal communities and interpersonal networks, higher levels of media use are associated with stronger relationships between religious activities and subjective wellbeing, possibly because modern media provides additional channels for communication, social connection, and identity expression. In contrast, in many European societies, religious institutions have historically provided relatively established sources of meaning and social integration. As modern media offers alternative channels for information, identity construction, and social interaction, greater media use may correspond to weaker associations between religious activities and subjective wellbeing in contexts where religion has historically provided established sources of meaning and social integration.

Overall, these findings indicate that modern media does not exhibit a uniform relationship with the religion–subjective wellbeing association. Instead, the observed relationship varies according to the compatibility between media-related cultural orientations and religious value systems. In contexts where modern media facilitates the reproduction of religious meanings and social belonging, the association between religious activities and subjective wellbeing tends to be stronger. Conversely, when media environments provide alternative or competing cultural frameworks, the association between religious activities and subjective wellbeing may become weaker. These results provide empirical support for the proposed cultural goal consistency framework.

### 4.4 Further analysis

This study further examines whether the association between religious activities and subjective wellbeing varies across different cultural contexts. Building on the cultural goal consistency perspective, this section analyses how macro-level national contexts, meso-level socioeconomic and educational characteristics, and micro-level generational differences are associated with variations in the relationship between religious activities and subjective wellbeing.

#### 4.4.1 Socioeconomic cultural differences

Individuals from different socioeconomic groups may hold different cultural orientations. Therefore, this study examines whether the relationship between religious activities and subjective wellbeing varies across income groups. In this study, the samples from China and Europe were, respectively, divided into four groups based on income levels: low income, lower-middle income, upper-middle income, and high income. The regression results are shown in results 1–4 of Table 7.

**Table 7:** The relationship between religious activities and subjective wellbeing at different levels.

Dimension		Row	China		Europe	
		(1)	Coeff.	*t*	Coeff.	*t*
Socioeconomic cultural	Low income	Result(1)	−0.0431	−1.00	0.1265***	11.08
	Lower-middle income	Result(2)	−0.1485*	−1.86	0.2441***	10.86
	Upper-middle income	Result(3)	−0.2538**	−2.15	0.3617***	10.74
	High income	Result(4)	−0.3591**	−2.29	0.4793***	10.67
Educational Cultural	Low education	Result(5)	0.0810**	2.17	0.0640***	7.56
	Lower-middle education	Result(6)	0.1016	1.57	0.1166***	7.21
	Upper-middle education	Result(7)	0.1223	1.30	0.1693***	7.04
	High education	Result(8)	0.1430	1.15	0.2219***	6.94
Age-related cultural	Elderly	Result(9)	0.1300***	3.15	0.1479***	15.77
	Older middle-aged	Result(10)	0.1991***	2.77	0.2839***	15.90
	Middle-aged	Result(11)	0.2682**	2.57	0.4199***	15.86
	Young people and children	Result(12)	0.3374**	2.45	0.5559***	15.82

The results suggest that the association between religious activities and subjective wellbeing differs across socioeconomic groups, and this pattern varies between China and Europe, supporting Hypothesis H4b.

The Chinese sample exhibits a “conflict intensification” pattern: the association between religious activities and subjective wellbeing is weaker among low-income groups and becomes statistically negative among middle- and high-income groups. This pattern may reflect differences in meso-level religious culture and macro-level mainstream culture. In the Chinese sample, religious culture has historically been embedded more strongly in community-based and interpersonal networks, representing a meso-level cultural resource, while broader social values provide the macro-level context associated with differences in the interpretation of religious meanings. Among higher-income groups, individuals may be more closely integrated into mainstream social institutions and cultural environments, which may correspond to differences in the extent to which religious values are perceived as compatible with broader social goals. Therefore, the weaker association between religious activities and subjective wellbeing among higher-income groups may reflect differences in cultural goal consistency across socioeconomic contexts.

The European sample exhibits a “synergistic association” pattern. A positive association was exhibited between religious activities and subjective wellbeing across income groups, with stronger associations among higher-income groups. One possible explanation is the compatibility of meso- and macro-level cultures in many European societies. European macro-level religion remains deeply compatible with macro-level regional culture (Christian tradition), although the degree of embeddedness varies considerably across European regions. Higher-income individuals may have greater access to social capital embedded in religious communities, which may correspond to stronger associations between religious participation and interpersonal support as well as cultural belonging.

#### 4.4.2 Educational cultural differences

Higher levels of education may be associated with differences in individuals’ interpretation of religious beliefs by increasing exposure to scientific knowledge, alternative value systems, and diverse sources of authority (Norris and Inglehart, 2011). This may correspond to differences in the associations between religious activities and subjective wellbeing. Therefore, participants were categorized by years of education into low, lower-middle, upper-middle and high educational attainment groups. The regression results are presented in results 4–8 of Table 7.

The results indicate that the association between religious activities and subjective wellbeing varies across educational groups, with different patterns observed between China and Europe, supporting Hypothesis H4c.

In the Chinese sample, the positive association between religious activities and subjective wellbeing is more pronounced among lower-educated groups, while this association becomes weaker among highly educated groups. This corroborates Norris and Inglehart’s theory: education provides alternative cultural systems, which may correspond to lower cognitive reliance on religion. In addition, within a social context where secular and non-religious value orientations are prominent, highly educated individuals may perceive greater distance between religious values and mainstream cultural orientations.

In European samples, the relationship between religious activities and subjective wellbeing remains relatively stable across educational groups. This may be because religious traditions in many European societies have historically coexisted with modern educational systems. Higher education may not necessarily correspond to weaker religious attachment but may instead facilitate more selective interpretations of religious beliefs, allowing individuals to maintain religious identity while adapting it to contemporary social values.

#### 4.4.3 Age-related cultural differences

People of different age groups have experienced different historical periods, such as the Cultural Revolution and the reform and opening up in China, and the Cold War and European integration in Europe. Individuals of different ages have perceived these phases differently, corresponding to age-related variations in cultural orientations. Participants were categorized into four age groups: children and young adults, middle-aged adults, older middle-aged adults, and older adults. The regression results are shown in results 9–12 of Table 7.

The results show that the relationship between religious activities and subjective wellbeing varies across age groups, supporting Hypothesis H4d. Age-related differences may reflect variations in cultural experiences and value internalization across the life course.

China and Europe exhibit a common “intergenerational experience–comfort association” pattern: the association between religious activities and subjective wellbeing is relatively stronger among younger individuals, while is weaker among middle-aged and elderly people. One possible explanation is that younger generations facing rapid social change and uncertainty may seek additional sources of meaning, identity, and social connection through cultural communities, including religious groups. For instance, church youth groups countering the atomization of society. For these individuals, religious activities may be associated with emotional support and a sense of belonging.

At the same time, the age-related micro-cultural differences does not completely eliminate the role of broader cultural contexts. For instance, elderly population in China experienced distinct historical and ideological contexts, epitomized by slogans such as “There has never been a savior, nor do we rely on gods or emperors.” Even so, religious activities can still be associated with psychological resources in the form of interpersonal bonds and emotional support. This indicates that micro-cultural cultural internalization may correspond to stronger associations than macro-level cultural constraints in certain contexts. When individuals face survival pressures, religion, as a meso-level group culture, may coexist with macro-cultural conflicts while remaining associated with psychological comfort.

Taking into account the associations across national, class, educational, and age cultural differences, this study identified a hierarchical pattern of cultural heterogeneity shown in Table 8. Macro-level cultural differences, including regional and ethnic cultural differences, are associated with variations in religious cultural adaptability. Mesocultural differences encompass group-based and class-based cultures, corresponding to variations in cultural adaptability. Microscopic cultural differences include individual cultural differences such as generational cultural differences, being associated with differences in the extent to which individuals accept and internalize religious culture. Among the three, the direction of mesocultural differences varies across macro-cultural contexts, while the role of macro-cultural conflicts appears to be less pronounced when microscopic cultural needs are strong.

**Table 8:** Cultural stratification regulation mechanism table.

Cultural levels	Regulatory function	Chinese model	European model
Macro (regional/ethnic)	Defining the boundaries of religious compatibility	Strong conflict (secular ethics vs. religion)	Strong compatibility (Christian tradition)
Middle view (group/class)	Deciding on cultural appropriateness filtering	Declining effect of high income/high education groups	The effect of high income/high education groups is increasing
Microcosm (the age of experience)	Controlling the degree of internalization of religious culture by individuals	Youth anxiety drives subcultural shift	Youth crisis drives return to tradition

## 5 Discussion

This study examined how religious activities and modern media use are associated with subjective wellbeing across China and European societies. The findings suggest that these relationships are not uniform but vary according to the cultural contexts in which individuals interpret and experience religious and digital environments. Rather than indicating universal benefits of either religion or media, the results highlight the importance of understanding how cultural environments are associated with differences in the relationship between different sources of meaning and connection and subjective wellbeing.

Consistent with previous evidence showing that religious activities are positively associated with subjective wellbeing (Lim and Putnam, 2010; Shiah et al., 2016), the present study further demonstrates that the strength of this association differs across social contexts. The stronger association observed in the Chinese sample suggests that religious participation may correspond to different psychological meanings and experiences in societies undergoing rapid social transformation. In such contexts, religious activities may be associated with interpersonal connection, emotional support, and meaning construction. In European societies, religious participation may operate more closely within established cultural institutions and community networks. These findings extend previous research by showing that the wellbeing relevance of religion varies with the social meanings attached to religious participation.

Beyond the contextual variation in religious participation, the findings further illustrate how digital transformation is associated with variations in the relationship between traditional meaning systems and individual wellbeing. Previous research has emphasized either the secularizing potential of digital media (Armfield and Holbert, 2003) or its capacity to facilitate religious communication and identity formation (Campbell and Tsuria, 2022). The present study contributes to this debate by showing that the relationship between media use and the wellbeing associations of religious activities is context dependent rather than characterized by a universal pattern of change in a single direction. In the Chinese sample, higher levels of media use are associated with a stronger positive association between religious activities and subjective wellbeing, possibly because digital platforms correspond to broader opportunities for emotional expression and community connection. In contrast, the negative interaction observed in the European sample suggests that, in contexts where religion already possesses institutional and cultural legitimacy, alternative value orientations disseminated through digital environments may correspond to a weaker association between religious participation and wellbeing.

Based on these differences, we further examined the heterogeneity across social socioeconomic, education and intergenerational groups. Previous studies have shown that religious meanings and practices are interpreted differently across socioeconomic groups, educational backgrounds, and generations, as individuals’ value orientations are associated with their social positions and historical experiences (Voas and Chaves, 2016; Thiessen and Wilkins-Laflamme, 2020). Consistent with this perspective, the present findings indicate that the wellbeing associations of religious activities vary depending on the extent to which religious meanings are compatible with individuals’ broader cultural environments. This extends previous research by highlighting the role of cultural goal consistency as a contextual condition associated with variations in religion–wellbeing relationships.

Furthermore, in increasingly digital societies, the coexistence of religious and digital meaning systems suggests that emerging cultural resources do not necessarily correspond to a decline in the relevance of traditional ones. Instead, their implications for subjective wellbeing vary according to how individuals integrate different sources of identity, belonging, and value orientations within specific cultural contexts (Effendi et al., 2026; Campbell and Evolvi, 2020).

## 6 Limitations and future research directions

Several limitations of this study should be acknowledged. First, the analysis is based on cross-sectional survey data from the CGSS and ESS, which limits the ability to draw causal inferences regarding the relationships between religious activities, media use, and subjective wellbeing. Although instrumental-variable estimation and robustness tests were conducted to address potential endogeneity concerns, future research should employ longitudinal or panel data to examine the dynamic relationships among these variables over time.

Second, despite efforts to improve comparability, the operationalization of religious activities and subjective wellbeing differs between the CGSS and ESS datasets due to variations in questionnaire design and cultural context. Although this study adopted independent regression strategies and logarithmic transformations to enhance comparability, measurement differences may still affect the interpretation of cross-context variations observed in specific settings.

Third, this study mainly focuses on religion and modern media as cultural connection channels, while other important cultural institutions such as family networks, local communities, and political organizations were not incorporated into the analytical framework. Future research could investigate how multiple cultural systems are associated with variations in subjective wellbeing under conditions of social transformation and digitalization.

## 7 Conclusion

This study explored how religious activities and modern media are jointly associated with subjective wellbeing across different cultural contexts. Using data from the CGSS and ESS, the findings suggest that both religion and modern media function as channels connecting individuals with systems of meaning, social identity, and cultural belonging. However, their associations with subjective wellbeing depend heavily on the consistency between religious cultural goals and broader social value orientations.

In an increasingly digital society, the cultural dimensions of subjective wellbeing should receive greater attention from policymakers, mental health professionals, and community organizations. Strengthening inclusive community networks, promoting healthy forms of online cultural participation, and enhancing individuals’ sense of meaning and belonging may contribute to social conditions associated with higher levels of psychological wellbeing under conditions of rapid social and technological change. In addition, the study highlights the importance of considering cultural compatibility when designing mental health interventions, digital communication strategies, and community development programs across different cultural contexts.

Overall, the relationship between religion and subjective wellbeing varies across cultural contexts rather than following a universal pattern in contemporary digital societies. Subjective wellbeing requires consideration of the interaction between cultural structures, technological environments, and individuals’ psychological needs for meaning, belonging, and emotional security.

## Data Availability

The original contributions presented in the study are included in the article/Supplementary material, further inquiries can be directed to the corresponding author.
