# Supplementary material for: Religious activities, modern media use, and subjective wellbeing: a cultural goal consistency perspective
Source: Front Psychol. 2026 Aug 28;17:1837670. doi: 10.3389/fpsyg.2026.1837670 (PMC13562042; doi:10.3389/fpsyg.2026.1837670)
Supplement: Supplementary file 1 [file Supplementary_file_1.DOCX]

Supplementary Material

# 1 Supplementary Figures and Tables

## 1.1 Supplementary Figures

**Figure 1.** Relationship between religion, Internet use and subjective well-being

## 1.2 Supplementary Tables

**Table 1.** Questions, options and assignments of control variables(China)

| Variable | Question | Options and value assigned | | | | | | | | | | | | | |
| --- | --- | --- | --- | --- | --- | --- | --- | --- | --- | --- | --- | --- | --- | --- | --- |
|  |  | 1 | 2 | 3 | 4 | 5 | 6 | 7 | 8 | 9 | 10 | 11 | 12 | 13 | 14 |
| Gender | respondent’s gender | Male | Female |  |  |  |  |  |  |  |  |  |  |  |  |
| Educational attainment | What is your highest level of education? | No education | Private tutoring | Literacy classes | Elementary school | Junior high school | Vocational high school | Regular high school | Secondary vocational school | Technical school | Associate degree (adult higher education) | Associate degree (regular higher education) | Bachelor's degree (adult higher education) | Bachelor's degree (regular higher education) | Graduate degree or higher |
| Income | What was your total income (in yuan) for the entire year of 2022? |  | | | | | | | | | | | | | |
| Political participation | What is your current political affiliation? | Masses | Communist Youth League member | Democratic party member | Communist Party member |  |  |  |  |  |  |  |  |  |  |
| Subjective health | How do you perceive your current physical health status? | Very unhealthy | Somewhat unhealthy | Average | Somewhat healthy | Very healthy |  |  |  |  |  |  |  |  |  |
| Objective health | In the past four weeks, how frequently have health issues affected your work or other daily activities? | Always | Often | Sometimes | Rarely | Never |  |  |  |  |  |  |  |  |  |
| Social interaction | Over the past four weeks, how often have you felt lonely? | Never | Rarely | Sometimes | Often | Very often |  |  |  |  |  |  |  |  |  |
| Objective social assessment | In the past year, have you frequently engaged in the following activities during your free time—socializing/ visiting others? | Never | Rarely | Sometimes | Often | Very frequently |  |  |  |  |  |  |  |  |  |
| Working hours | When you have a job, how many hours do you typically work per week, including overtime? |  | | | | | | | | | | | | | |

**Table 2.** Questions, options and assignments of control variables(Europe)

| Variable | Question | Options and value assigned | | | | | | | | | |
| --- | --- | --- | --- | --- | --- | --- | --- | --- | --- | --- | --- |
|  |  | 1 | 2 | 3 | 4 | 5 | 6 | 7 | 8 | 9 | 10 |
| Gender | Which of the options on this card best describes you? | Male | Female |  |  |  |  |  |  |  |  |
| Educational attainment | About how many years of education have you completed, whether full-time or part-time? |  | | | | | | | | | |
| Income | Using this card, please tell me which letter describes your household’s total income, after tax and compulsory deductions, from all sources? | J | R | C | M | F | S | K | P | D | H |
| Political participation | Have you donated to or participated in a political party or pressure group？ | No | Yes |  |  |  |  |  |  |  |  |
| Subjective health | How is your health in general? | Very bad | Bad | Fair | Good | Very good |  |  |  |  |  |
| Objective health | Are you hampered in your daily activities in any way by any longstanding illness, or disability, infirmity or mental health problem? | Yes, a lot | Yes, to some extent | No |  |  |  |  |  |  |  |
| Subjective loneliness perception | How much of the time during the past week did you feel this wayyou felt lonely? | None or almost none | Sometimes | Most of the time | All or almost all of the time |  |  |  |  |  |  |
| Objective social assessment | Compared to other people of your age, how often would you say you take part in social activities? | Much less than most | Less than most | About the same | More than most | Much more than most |  |  |  |  |  |
| Working hours | Regardless of your basic or contracted hours, how many hours {do/did} you normally work a week (in your main job), including any paid or unpaid overtime |  | | | | | | | | | |

**Table 9.** Cultural Stratification Regulation Mechanism Table

| Cultural Levels | regulatory function | Chinese model | European model |
| --- | --- | --- | --- |
| Macro (regional/ethnic) | Defining the boundaries of religious compatibility | Strong conflict (secular ethics vs. religion) | Strong compatibility (Christian tradition) |
| Middle view (group/class) | Deciding on cultural appropriateness filtering | Declining effect of high income/high education groups | The effect of high income/high education groups is increasing. |
| Microcosm (The Age of Experience) | Controlling the degree of internalization of religious culture by individuals | Youth anxiety drives subcultural shift | Youth crisis drives return to tradition |
